# Comparative genomics of *Salmonella enterica* serovar Enteritidis ST-11 isolated in Uruguay reveals lineages associated with particular epidemiological traits

**DOI:** 10.1038/s41598-020-60502-8

**Published:** 2020-02-27

**Authors:** Bruno D’Alessandro, Victoria Pérez Escanda, Lucía Balestrazzi, Florencia Grattarola, Andrés Iriarte, Derek Pickard, Lucía Yim, José Alejandro Chabalgoity, Laura Betancor

**Affiliations:** 1 Departamento de Desarrollo Biotecnológico, Instituto de Higiene, Facultad de Medicina, Universidad de la República, Av. Alfredo Navarro 3051, CP 11600 Montevideo, Uruguay; 2 Departamento de Bacteriología y Virología, Instituto de Higiene, Facultad de Medicina, Universidad de la República, Av. Alfredo Navarro 3051, CP 11600 Montevideo, Uruguay; 3 Wellcome Trust Sanger Institute, Wellcome Trust Genome Campus, Hinxton, Cambridge, UK

**Keywords:** Phylogenetics, Bacterial genomics

## Abstract

*Salmonella enterica* serovar Enteritidis is a major cause of foodborne disease in Uruguay since 1995. We used a genomic approach to study a set of isolates from different sources and years. Whole genome phylogeny showed that most of the strains are distributed in two major lineages (E1 and E2), both belonging to MLST sequence type 11 the major ST among serovar Enteritidis. Strikingly, E2 isolates are over-represented in periods of outbreak abundance in Uruguay, while E1 span all epidemic periods. Both lineages circulate in neighbor countries at the same timescale as in Uruguay, and are present in minor numbers in distant countries. We identified allelic variants associated with each lineage. Three genes, *ycdX*, *pduD* and *hsdM*, have distinctive variants in E1 that may result in defective products. Another four genes (*ybiO*, *yiaN*, *aas*, *aceA*) present variants specific for the E2 lineage. Overall this work shows that *S*. *enterica* serovar Enteritidis strains circulating in Uruguay have the same phylogenetic profile than strains circulating in the region, as well as in more distant countries. Based on these results we hypothesize that the E2 lineage, which is more prevalent during epidemics, exhibits a combination of allelic variants that could be associated with its epidemic ability.

## Introduction

*Salmonella* is a major cause of human foodborne disease worldwide. A singular epidemiological feature of human salmonellosis is that one particular serovar can become prevalent over the others, but the prevalent serovar may change over time. Previous to the 1980’s, *Salmonella enterica* serovar Typhimurium was the most commonly isolated serovar worldwide, but then at the beginning of the 1990’s *S*. *enterica* serovar Enteritidis emerged as the most common cause of human salmonellosis, first in Europe and then in many other countries arround the world^1–6^. The reasons for this serovar shift are still not fully understood.

Several studies have addressed the phylogenetic diversity within S. *enterica* serovar Enteritidis, and suggested that diversification events could be related to its epidemiological features^7–10^. The work of Allard *et al*. first reported the use of whole genome sequencing (WGS) and single nucleotide polymorphisms (SNP) analysis to address molecular epidemiology of a set of isolates previously indistinguishable by other techniques. Deng *et al*. suggested that serovar Enteritidis diversified from a few major lineages spread worldwide, and associated some lineages with geographic and epidemiological characteristics. Feasey *et al*. found a strong correlation between prophage content and accessory genome features with the establishment of a new epidemiological course. In our studies, we found that the acquisition of a new prophage was probably determinant for the onset of a particular lineage^8^. The vast majority of *S*. *enterica* serovar Enteritidis isolates worldwide belong to the eBurst Group 4 (EBG4). Achtman *et al*. and more recently our group described that among EBG4, multi locus sequence type (MLST) ST11 is the major ST and constitutes a polyphyletic lineage from which several other minor lineages emerged^8,11,12^.

**Figure 1:**
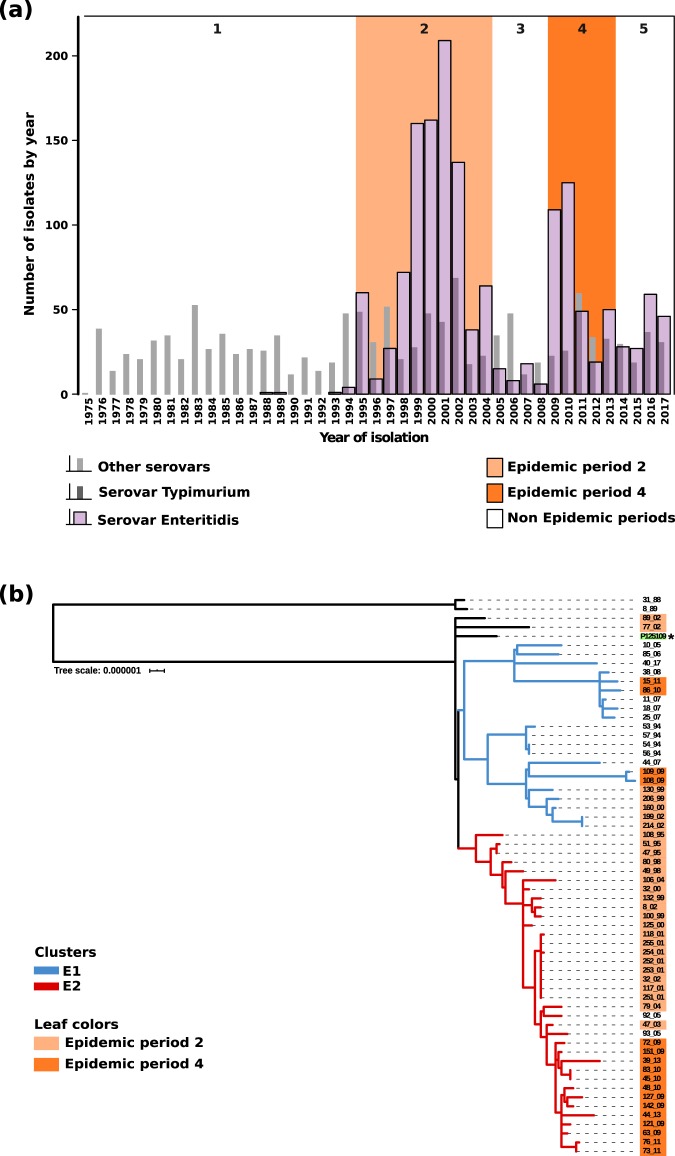
(**a**) Distribution of *S*. *enterica* serovars received at the National *Salmonella* Center over the time (1975 to 2017). The 5 epidemiological periods of isolation are indicated with numbers. In white: non epidemic periods (1, 3 and 5). In orange: epidemic periods (2 and 4). (**b**) Phylogenetic tree showing the two clusters among Uruguayan *S*. *enterica* serovar Enteritidis genomes (E1 in blue, E2 in red and other strains in black). Highlight over the strain designation indicate the period of isolation as explained above. The genome of reference (P125109) is highlighted in green and marked with an asterisk. The isolates are named using a number followed by an underscore and the last two digits of the year of isolation. Scale bar units represents changes per variant site.

In Uruguay, until 1994 *S*. *enterica* serovar Enteritidis was only sporadically isolated, but in 1995 an important outbreak of human salmonellosis associated to this serovar occurred. Since then it became the most prevalent serovar in the country despite some periodical declining^1^. Five different epidemiological periods can be distinguished, as related to the prevalence of serovar Enteritidis in the country: a pre-epidemic period, the first epidemic (1995–2004), a declining (2005–2008), a second epidemic period (2009–2013) and further declining afterward (see Fig. 1(a)).

In previous studies, we found that the two oldest pre-epidemic isolates (31/88 and 8/89) belong to a unique genetic type by RAPD, PFGE, and MLST and have impaired behavior in different models of infection^1,8,13^. In spite of these observations, we could not find a correlation between genetic features and different pathogenic or epidemiological abilities among the isolates. In the present study, we analyze the genomic sequences of 61 *S*. *enterica* serovar Enteritidis strains isolated in Uruguay between 1988 and 2017 from different sources by comparative genomics and SNP based phylogenetic analysis. We extended the study to include strains from Argentina and Brazil. An analysis of the genomic differences between the lineages that have been circulating since the beginning of the *S*. *enterica* serovar Enteritidis epidemic period is presented.

## Methods

### Bacterial strains, culture and DNA extraction

A total of 61 *S*. *enterica* serovar Enteritidis strains isolated in Uruguay from different sources and periods were selected from the collection of the National *Salmonella* Center (Instituto de Higiene, Universidad de la República). All strains were previously characterized by our group using different genetic and phenotypic methods^13,14^. Strain names, source and date of isolation are shown in Supplementary Table 1. The strains were recovered from freeze stocks (kept at −80 °C in LB broth with 16% glycerol) by culture in LB agar (Sigma-Miller). Liquid cultures used for DNA extraction were performed in LB broth at 37 °C with shaking. DNA extraction and purification were performed using commercial purification kits (DNeasy Blood & Tissue Kit, Qiagen).

### Genome sequencing and assembly

Genome sequencing was performed using Illumina technology, and the sequences were deposited in the NCBI database. The total number of produced paired-end reads for each strain ranged from 0.66 × 10^6^ to 7.9  × 10^6^, with a read size of 100, 277 and 76 for platforms Hiseq. 2000, MySeq v3, and Illumina Genome Analyzer II, respectively. Generated reads were trimmed using sickle (available at github.com/najoshi/sickle), with a phred score threshold of 30 (Q30). Quality was finally checked using FastQC (www.bioinformatics.babraham.ac.uk/projects/fastqc/). For platform and sequencing details see Supplementary Table 1. *De novo* assembly was performed with Spades (version 3.6.1)^15^ using a pre-assembly approach with Velvet (version 1.2.10)^16^. The first assembly was done using Velvet with default parameters. The final assembly was performed in Spades using a range of k-mer sizes between 29 and the maximum read size minus one. Velvet-generated contigs were used as a reference with the “--untrusted-contigs” option. On average 97.7% of the generated reads were mapped and the mean observed nucleotide coverage ranged from 27 to 254×. N50 contig size has an average size of 447.312. Assembly stats are summarized in Supplementary Table 1. All the Uruguayan genomes are available in the NCBI BioProject database with accession numbers PRJEB2130 and PRJEB4649 (Supplementary Table 1).

### Comparative genomics

The genome of phage type 4 strain P125109, (GenBank ID AM933172.1, NCBI refseq accession NC_011294.1), was used as reference when needed. All the genomic sequences were annotated using RASTtk^17^ and verified with EnteroBase annotation pipeline. Comparative SNP analysis was performed using NUCmer (NUCleotide MUMmer version 3.1) and local blast (version 2.2.30)^18^. NUCmer was run with the parameters “-maxmatch” and the minimum length of a single exact match set to 12 (-l parameter). SNPs were obtained using the show-snps module with the –C option, so excluding all SNPs from repeats.

Phylogeny figures were generated using iTOL^19^. Figures showing the alignment between variants of genes were generated through BLASTn alignments using EasyFig^20^, exported to svg images and edited using Inkscape software (inkscape.org).

For phylogenetic analysis all fastq files were submitted to EnteroBase which automatically performs a pipeline of assembly, annotation and some *in silico* typing schemes including Achtman 7 gene MLST and ribosomal MLST (rMLST) (http://enterobase.warwick.ac.uk)^12^. EnteroBase tools were used to perform a whole genome SNP phylogeny, using the genome of strain P125109 as reference. The EnteroBase algorithm performs *de novo* assembly from reads and uses LAST software to call the SNPs from the assembled genomes, filters out SNPs from repetitive regions and low quality variants, and builds a SNP matrix which is used as input to perform a RAxML maximum likelihood phylogeny^21^. For the SNP tree we considered only the sites present in 100% of the genomes.

Genomes from strains isolated in Argentina and Brazil that were included in phylogenetic analysis are shown in Supplementary Table 2 and were selected from the available genomes in EnteroBase.

## Results and Discussion

### Comparative genomics

First we compared the 61 Uruguayan isolates using SNP analysis with the P125109 genome as reference. This analysis showed that the two oldest isolates have more than 600 SNPs of difference compared to the reference genome (607 and 652 for 31/88 and 08/89 respectively), whereas all other 59 isolates have less than two hundred SNPs (from 60 to 166) (Supplementary Table 1). On the other hand, all Uruguayan genomes share 17 SNPs of difference with P125109 genome.

A phylogenetic analysis based on SNPs revealed the existence of two clades that we named E1 and E2 (Fig. 1(b)) that comprise 57 out of the 61 strains. These 57 strains belong to the major *S*. *enterica* serovar Enteritidis MLST sequence type 11 (ST-11) and have been co-circulating in the country since 1994. The remaining four isolates (31/88, 8/89, 77/02 and 89/02) did not group with any of the two clades (Fig. 1(b)). We previously reported that the two oldest isolates (31/88 and 8/89) belong to the MLST sequence type 1974 (ST1974) and that ST1974 originated from ST11 after the acquisition of a characteristic prophage^8^. The other two isolates, 77/02 and 89/02, belong to ST11 as the majority of the strains.

The E1 clade comprises 21 strains that span all five epidemiological periods, and were isolated from food, animal or human infections. Instead, E2 clade is represented by 36 isolates of which 34 were isolated in the periods associated to epidemics (Fig. 1(a,b)). Hence, it is reasonable to hypothesize that E2 may constitute a lineage that was responsible for the *S*. *enterica* serovar Enteritidis epidemics in the country.

Previously we reported a phylogenetic analysis of 203 strains, representing intra-serovar diversity among *S*. *enterica* serovar Enteritidis EBG4 isolates worldwide, that included 6 of the 61 Uruguayan isolates used in the present study^8^. As we found now, half of these 6 strains are E1 and half are E2 (E1: 53/94, 206/99 and 214/02; E2: 8/02, 251/01 and 253/01), and each of these two groups is closely related with strains from Europe and North America. This relation is showed in Supplementary Fig. S1, containing the previously reported phylogeny with a zoom on the clade formed by these strains. This suggests that E1 and E2 may have a broad circulation pattern.

**Figure 2:**
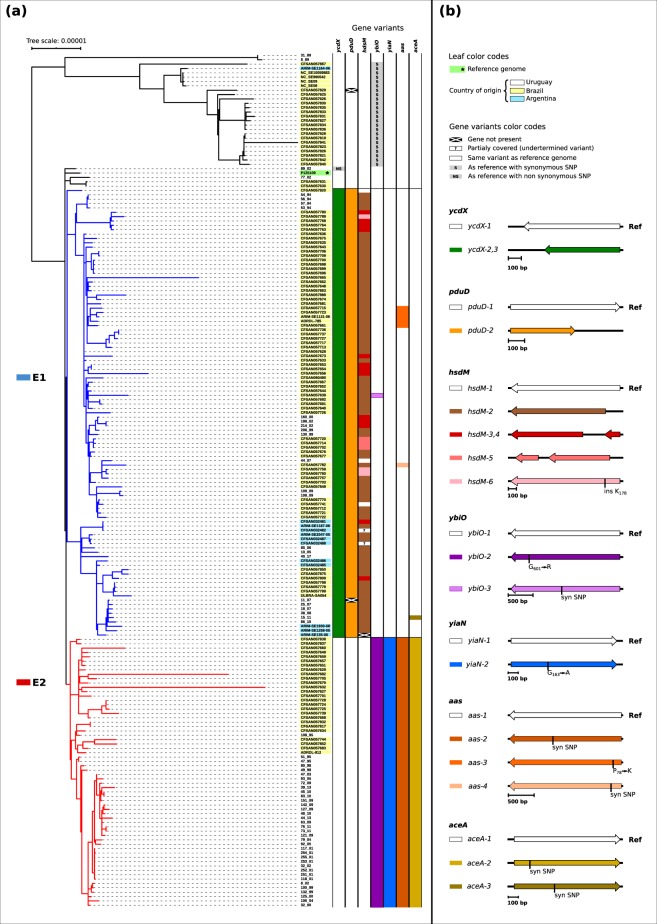
(**a**) Phylogenetic tree of the 195 genomes including 61 from Uruguay (white leaves), 12 from Argentina (light blue leaves) and 122 from Brazil (yellow leaves). Branches of E1 lineage are colored in blue. Branches of E2 lineage are indicated in red. Lanes at right represent with different colors the allelic variants for *ycdX*, *pduD*, *hsdM*, *ybiO*, *yiaN*, *aas* and *aceA* respectively. See colour legends inside the (**b**). Scale bar units represents changes per variant site. (**b**) Gene alignments for the different allelic variants of *ycdX*, *pduD*, *hsdM*, *ybiO*, *yiaN*, *aas* and *aceA* respectively. Genes are depicted as annotated in the genome containing each variant and aligned to the reference P125109. The scale of the alignment in base pairs (bp) is indicated by horizontal bars. SNPs associated to each variant respect to the reference genome are detailed in Table 1. SNPs causing non synonymous variants are marked as X → Y (in single letter aminoacid code) relative to its position in the gene. Synonymous SNPs are only marked relative to its position in the gene (syn SNP).

In order to further support this assumption we performed a new phylogenetic analysis, this time with focus in strains isolated in the region, including 12 from Argentina, 122 from Brazil and the same 61 genomes from Uruguay (Fig. 2(a)). Interestingly we found that all these strains clustered in a highly similar way of what we found among Uruguayan isolates, i.e. E1 cluster now contains strains from Argentina and Brazil whereas E2 cluster contains strains from Brazil (103 in E1, and 62 in E2). Given the low number of genomes from Argentina available in EnteroBase, we can not exclude the possibility that lineage E2 was circulating in this country. Only 30 strains from all three countries grouped outside E1 and E2, of which 24 were isolated before 1994 (Fig. 2(a), Supplementary Table 2). All in all our results strongly suggest that E1 and E2 strains were introduced in the region arround 1994 and have been circulating ever since in Uruguay, Brazil, Argentina, Europe and North America, supporting the idea that both constitute lineages of the serovar Enteritidis.

We then performed a comparative genomics analysis aimed to identify genetic differences associated with lineages E1 and E2, including 165 genomes: 57 from Uruguay, 11 from Argentina and 97 from Brazil (Fig. 2(a)).

### Allelic variants among E1 and E2 lineages

As we did not find differences in accessory genome between E1 and E2 lineages, we looked for SNP differences among the 165 selected strains. This analysis showed that the main differences between E1 and E2 are located in three genes: *ycdX*, *pduD* and *hsdM*. These genes are interrupted in E1 genomes when compared to their counterparts in the E2 genomes. In addition, we found allelic variants in other four genes, *ybiO*, *yiaN*, *aas and aceA*, that are specific for each lineage (Fig. 2(a,b); Table 1).

**Table 1:** SNPs and gene variants of the 7 gene markers for the lineages E1 and E2.

Gene	Variant name	SNP	E1	E2	Observations
*ycdX*	ycdX-1	—	0%	100%	
	ycdX-2	(-→ AGCC) _552_	99%	0%	Frameshift _187_; stop _193_
	ycdX-3	(- → CCAG) _552_	1%	0%	Frameshift _187_; stop _193_
*pduD*	pduD-1	—	0%	100%	
	pduD-2	(C→ T)_396_	99%	0%	Stop _133_
*hsdM*	*hsdM-1*	—	3%	100%	
	hsdM-2	(- → T) _171_	77%	0%	Frameshift _60_; stop _64_; late start CDS.
	hsdM-3	(- →TT) _171_	13%	0%	Frameshift _60_; stop _60_ and CDS in 2nd frame
	hsdM-4	(- → T)_178_; (G → T) _797_	1%	0%	Frameshift _60_; stop _64_ and CDS in 2nd frame
	hsdM-5	(- → T)_178_;(T → -) _867_	3%	0%	Frameshift 60; stop 64; late start and truncated CDS.
	hsdM- 6	(- → TTT) _178_	3%	0%	insert Lys _59_
*ybiO*	ybiO-1	—	98%	0%	
	ybiO-2	(T → G) _1803_	0%	100%	Gly _601_ ->Arg
	ybiO-3	(G → A) _1125_	2%	0%	Synonymous
*yiaN*	yiaN-1	—	100%	0%	
	yiaN-2	(G → C) _489_	0%	100%	Gly _163_ ->Ala
*aas*	aas-1	—	94%	0%	
	aas-2	(T → C) _1301_	0%	100%	Synonymous
	aas-3	(C → T) _233_	5%	0%	(Pro_78_ → Lys)
	aas-4	(G → A) _270_	1%	0%	Synonymous
*aceA*	aceA-1	—	99%	0%	
	aceA-2	(C → T) _198_	0%	100%	Synonymous
	aceA-3	(G → A) _543_	1%	0%	Synonymous

The variant names correspond to those showed in Fig. 2(b). SNP column show the nucleotidic change respect to the reference genome in brackets (bases as in the leading strand of the reference genome P125109) and its relative position to the gene start in subscript. Columns E1 and E2 indicate the percentage of genomes that present the corresponding variant in each lineage. Note that the total number of E1 genomes is 103 and the total number of E2 genomes is 62.

All the 103 E1 strains (11 from Argentina, 71 from Brazil and 21 from Uruguay) show a 4 base pair insertion located in gene *ycdX* (SEN1912) as compared to the reference and to all E2 strains (Table 1). This insertion introduces a premature stop codon, which may produce a pseudo-gene or a truncated version of the protein (Fig. 2(b) and Table 1). The *ycdX* gene encodes an hydrolase that belongs to the superfamily of PHP proteins, that contain a NH2-terminal php domain (polymerase histidinol phosphatase). This domain is characteristic of the DNA polymerase III alpha subunit of bacteria and is involved in the proofreading function^22^. It has been suggested that YcdX is involved in the DNA repair pathways in *E*. *coli* and could act as a nuclease or a phosphatase in these pathways^23^. Recently, Wang *et al*. found two allelic variants for this gene (non synonymous SNP) between *S*. *enterica* serovar Enteritidis isolates with markedly different abilities to survive in egg white^24^. Inoue *et al*. showed that mutation in *ycdY* (the adjacent gene in the *ycdWXYZ* operon) caused repression of swarming in *E*. *coli*^25^.

All the strains from the E2 lineage have the same allelic variant for *pduD* gene (SEN2039) while 102 out of the 103 strains from E1 lineage show a base substitution that introduce a stop codon, similar to the one observed for *ycdX* (Table 1). The remaining strain (i.e. 11/07 from Uruguay) completely lacks the *pduD* gene (Fig. 2(b) and Table 1). *pduD* encodes for a propanediol dehydratase, part of the *pdu* operon which encodes all the required proteins for the 1,2-propanediol catabolism. This operon that was acquired by lateral gene transfer in *Salmonella*^26,27^ is disrupted in different serovars that cause invasive extra-intestinal infections in human such as Typhi and Paratyphi A amongst others^28^. Faber *et al*. reported that the functionality of this operon can be important to compete with intestinal microbiota, as 1,2 propanediol is a metabolic product in the anaerobic gut environment that can be used by *Salmonella* when coupled to the tetrathionate anaerobic respiration^29^. It is proposed that ability to use 1,2 propanediol allow the expansion of the pathogen population in the gut increasing the excretion in feces, which is an important factor for epidemic dissemination^29^.

Furthermore, all E2 strains have the same allelic variant for the *hsdM* gene (SEN4290) but among the E1 strains there are several variants for this gene (Fig. 2(b) and Table 1). Most of the E1 strains contain variants of *hsdM* that would produce either a truncated version (4 out of the 6 variants) or a non synonymous substitution for the protein. Only three strains in the E1 (including the Uruguayan 44/07) have the same variant as E2, suggesting that only in E1 strains *hsdM* is prone to accumulate mutations (Fig. 2(b)). The *hsdM* gene encodes a DNA methylase involved in type I restriction-modification system (RM) in enterobacteria^30,31^. Previous reports linked restriction modification systems type 2 with pathogenicity in *Salmonella* due to epigenetic effects on the virulence gene expression^32^. Silva *et al*. showed that mutations in the RM genes produce an impaired phenotype in the mouse model of invasive salmonellosis^33^. Type 1 RM systems were also described as involved in pathogenicity of *Yersinia pseudotuberculosis*^34^.

Given that E2 but not E1 have intact copies of *ycdX*, *pduD* and *hsdM*, and considering that these genes have been previously implicated in *Salmonella* pathogenicity, it can be taken to suggest that these three genes may be relevant for the epidemic ability of the E2 strains. We also looked for the allelic variants in the global phylogeny representing the intra-serovar diversity (Supplementary Fig. 1 and ref. ^8^) and found that the predominant allele is the E2 allele in all cases, whereas the disrupted variants are specific for E1 (data not shown).

As mentioned above, another four genes *ybiO*, *yiaN*, *aas* and *aceA* were found with minor sequence differences between both lineages. These four genes present a single variant in E2 strains whereas E1 are mostly identical to P125109 reference strain.

All E2 strains present a particular allelic variant of the *ybiO* gene (SEN0772) due to a substitution that produce a Gly(601)→Arg in the protein (Fig. 2(a,b), Table 1). The *ybiO* gene encode for a putative mechanosensitive channel protein involved in osmotic adaptation that is activated by hypo-osmotic shock in *E*. *coli*^35^. It was previously reported that there are two allelic variants for this gene among *S*. *enterica* serovar Enteritidis isolates that have differing ability to survive in egg white^24^. For *yiaN* (SEN3494) (L-dehydroascorbate transporter large permease subunit, a putative membrane protein) all E2 genomes present the same allele that differs in Gly(163)→Ala respect to E1. Similarly, all E2 share the same variant for *aas* (2-acylglycerophosphoethanolamine acyltransferase/acyl-ACP synthetase, SEN2853) and *aceA* (isocitrate lyase, SEN3966) genes. In E1, most of the strains have an aas *gene* identical to the reference, and a few strains present either a non synonymous substitution (5 strains) or a synonymous substitution (1 strain) (Fig. 2(a), Table 1). Similarly, the *aceA* gene is identical to the reference in all but one of the E1 genomes.

In summary, we found that the E2 alleles for *ybiO*, *yiaN*, *aas* and *aceA* are specific for this lineage.

## Conclusions

Two different lineages have been circulating in Uruguay after the start of the epidemic period. These same two lineages also circulated in Brazil in similar periods of time as well as in Europe and North America. Our results are in agreement with those recently published by Campioni *et al*. that report the existence of a shift in the genomic profile of *S*. *enterica* serovar Enteritidis circulating in South America after 1994^36^. Based on all these results, we hypothesize that around 1994 phage type 4 strains (likely from E1 and E2 lineages) were introduced in the region and successfully spread causing epidemics. These introduced strains belong to one of the sub-clades described by Feasey as part of the global epidemic *S*. *enterica* serovar Enteritidis clade^10^.

Further, as reported here, at least in Uruguay, the E2 lineage is clearly associated with epidemics. This lineage presents a combination of allelic variants that could be associated with its epidemic ability. The fact that genes *ycdX*, *pduD* and *hsdM* are disrupted in E1 strains that come from different regions and periods of isolation is suggestive of a biological relevance for conserving this disruption. The disrupted version of these genes is a genetic marker for the E1 lineage, and we propose that it might be related to impaired epidemic ability. On the other hand, the allelic variants of another four genes (*ybiO*, *yiaN*, *aas*, *aceA*) are specific markers for the E2 lineage, and could be useful for tracking the presence of this lineage in further epidemic events.

## Supplementary information

Supplementary Figure 1.

Supplementary tables 1 and 2.

## Data Availability

All the Uruguayan genomes are available in the NCBI BioProject database with accession numbers PRJEB2130 and PRJEB4649, with the specific accession for each sample showed in Supplementary Table 1. The accession numbers for all the genomes from regional strains are showed in Supplementary Table 2.
