# Publisher Correction: Comparative genomics of *Salmonella enterica* serovar Enteritidis ST-11 isolated in Uruguay reveals lineages associated with particular epidemiological traits

**DOI:** 10.1038/s41598-020-63863-2

**Published:** 2020-05-13

**Authors:** Bruno D’Alessandro, Victoria Pérez Escanda, Lucía Balestrazzi, Florencia Grattarola, Andrés Iriarte, Derek Pickard, Lucía Yim, José Alejandro Chabalgoity, Laura Betancor

**Affiliations:** 1 Departamento de Desarrollo Biotecnológico, Instituto de Higiene, Facultad de Medicina, Universidad de la República, Av. Alfredo Navarro 3051, CP 11600 Montevideo, Uruguay; 2 Departamento de Bacteriología y Virología, Instituto de Higiene, Facultad de Medicina, Universidad de la República, Av. Alfredo Navarro 3051, CP 11600 Montevideo, Uruguay; 3 Wellcome Trust Sanger Institute, Wellcome Trust Genome Campus, Hinxton, Cambridge UK

Correction to: *Scientific Reports* 10.1038/s41598-020-60502-8, published online 27 February 2020

This Article contains an error in Figure 1 where,

‘Serovar Typimurium’

should read:

‘Serovar Typhimurium’

In addition, the bars corresponding to Serovar Typhimurium are missing.

The correct Figure 1 appears below.

**Figure 1:**
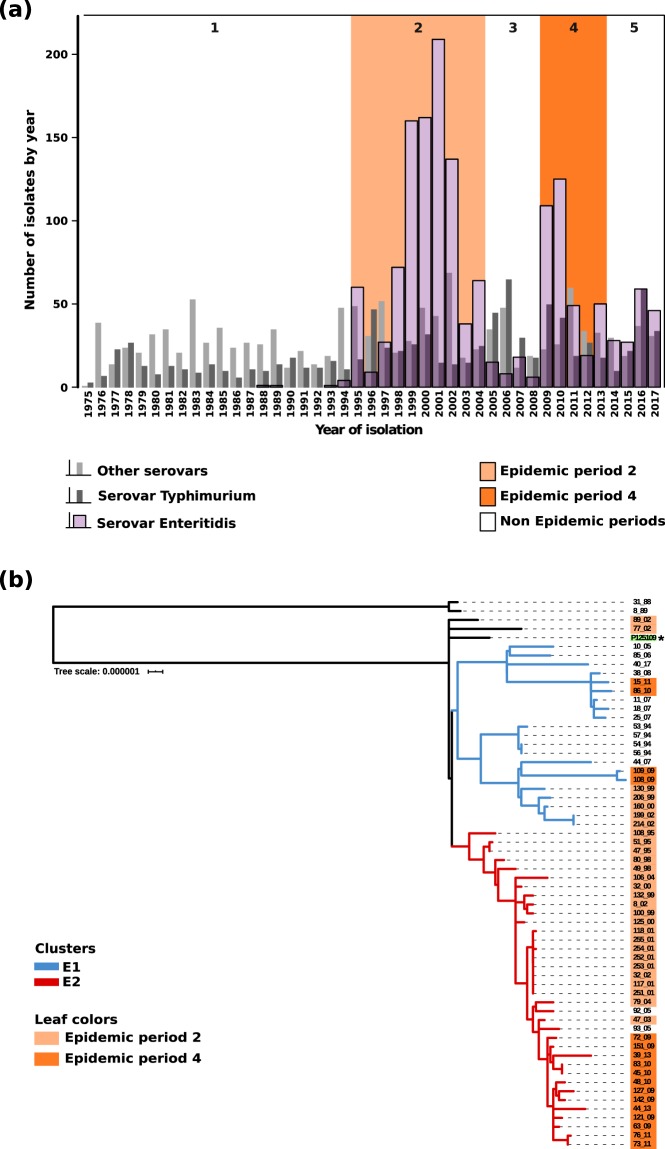
.

