# Supplementary material for: Comparative genomics of Salmonella enterica serovar Enteritidis ST-11 isolated in Uruguay reveals lineages associated with particular epidemiological traits
Source: Sci Rep. 2020 Feb 27;10:3638. doi: 10.1038/s41598-020-60502-8 (PMC7046640; doi:10.1038/s41598-020-60502-8)

Comparative genomics of *Salmonella enterica* serovar Enteritidis ST-11 isolated in Uruguay reveals lineages associated with particular epidemiological traits.

Bruno D'Alessandro [1],
Victoria Pérez Escanda [1],
Lucía Balestrazzi [1]
Florencia Grattarola [2]
Andrés Iriarte [1],
Derek Pickard [3],
Lucía Yim [1],
José Alejandro Chabalgoity [1],
*Laura Betancor** [1,2]

1- Departamento de Desarrollo Biotecnológico, Instituto de Higiene, Facultad de Medicina, Universidad de la República, Montevideo, Uruguay; Av. Alfredo Navarro 3051, CP 11600.
2- Departamento de Bacteriología y Virología, Instituto de Higiene, Facultad de Medicina, Universidad de la República, Montevideo, Uruguay; Av. Alfredo Navarro 3051, CP 11600.
3- The Wellcome Trust Sanger Institute, The Wellcome Trust Genome Campus, UK

* *Corresponding author* laurabet@higiene.edu.uy.

Supplementary Figure 1S

This figure shows the E1 and E2 clusters in the eBG4 phylogeny using a selection of genomes representing the serovar Enteritidis diversity (8). The sequence type (ST) of the genomes is indicated at rigth. The region corresponding the E1 and E2 clusters (yellow box) is zoomed from the original tree and showed at left in the dotted box. The blue branches correspond to E1 and the red branches to E2. With the exception of the Uruguayan genomes (referred here only by their isolate name), the leaves in the zoomed area show this information in order: i) the corresponding leave number from the main tree (in brackets), ii) strain name, iii) country, and iv) year of isolation. Detailed information about the methods and genomes used to generate this tree should be obtaned in D'Alessandro et al 2018 (8).

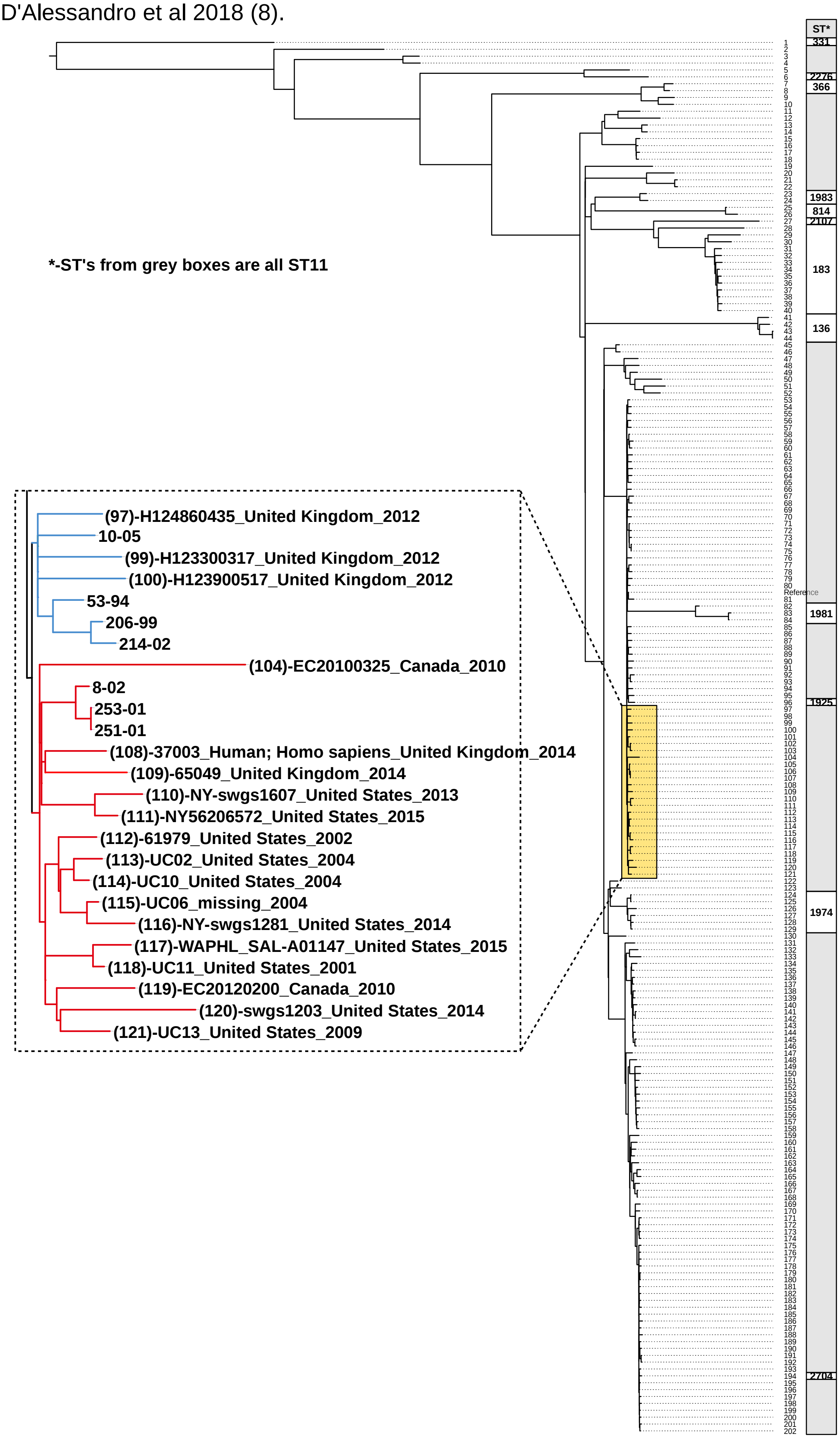

Supplement: Supplementary file 1 — Supplementary Figure 1. [file 41598_2020_60502_MOESM1_ESM.pdf]
